# Tree Nut Consumption Is Associated with Better Nutrient Adequacy and Diet Quality in Adults: National Health and Nutrition Examination Survey 2005–2010

**DOI:** 10.3390/nu7010595

**Published:** 2015-01-15

**Authors:** Carol E. O’Neil, Theresa A. Nicklas, Victor L. Fulgoni

**Affiliations:** 1 Louisiana State University Agricultural Center, Baton Rouge, LA 70803, USA; 2 USDA/ARS Children’s Nutrition Research Center, Department of Pediatrics, Baylor College of Medicine, Houston, TX 77030, USA; E-Mail: tnicklas@bcm.edu; 3 Nutrition Impact, LLC, Battle Creek, MI 49014, USA; E-Mail: VIC3RD@aol.com

**Keywords:** tree nuts, NHANES, adults, nutrient adequacy, diet quality

## Abstract

Nutrient adequacy of tree nut consumers has not been examined. The National Health and Nutrition Examination Survey 2005–2010 data were used to assess the association of tree nut consumption by adults 19+ years (*n* = 14,386) with nutrient adequacy and diet quality. Covariate adjusted usual intake was determined using two 24-h dietary recalls and the National Cancer Institute method. Percentages of the consumption groups below the Estimated Average Requirement (EAR) or above the Adequate Intake (AI) were determined. Diet quality was determined using the Healthy Eating Index-2005 (HEI) score. Usual intake data showed consumers of tree nuts had a lower percentage (*p* < 0.0001) of the population below the EAR for vitamins A (22 ± 5 *vs.* 49 ± 1), E (38 ± 4 *vs.* 94 ± 0.4) and C (17 ± 4 *vs.* 44 ± 1); folate (2.5 ± 1.5 *vs.* 12 ± 0.6); calcium (26 ± 3 *vs.* 44 ± 1); iron (3 ± 0.6 *vs.* 9 ± 0.4); magnesium (8 ± 1 *vs.* 60 ± 1); and zinc (1.5 ± 1 *vs.* 13 ± 1). Tree nut consumers had a higher percentage (*p* < 0.0001) of the population above the AI for fiber (33 ± 3 *vs.* 4 ± 0.3) and potassium (12 ± 3 mg *vs.* 2 ± 0.2 mg). HEI-2005 total score was higher (*p* < 0.0001) in tree nut consumers (61 ± 0.7 *vs.* 52 ± 0.3) than non-consumers. Health professionals should encourage the use of tree nuts as part of a dietary approach to healthy eating.

## 1. Introduction

Tree nuts have been consumed since the pre-historic era and have provided a concentrated source of energy and nutrients to consumers [1]. The nutrient content of tree nuts varies by species, but in general they are rich sources of energy, vegetable protein, dietary fiber, vitamins E and K, folate, magnesium, copper, and potassium. Some tree nuts are especially good sources of specific nutrients, for example almonds are high in vitamin E and Brazil nuts are high in selenium [2]. The lipid profile of most tree nuts also supports cardiovascular health [3,4,5,6], in that tree nuts are cholesterol free and high in mono-(MUFA) or high in poly-unsaturated fatty acids (PUFA) and low in saturated fatty acids [2]. All tree nuts are naturally low in sodium [2].

Previous studies using data from the National Health and Nutrition Examination Survey (NHANES) have shown that adults who consumed tree nuts [7,8,9,10] and all nuts (includes peanuts—a legume) [9] had better nutrient intakes than non-consumers. O’Neil *et al.* [7] showed that consumers of tree nuts had significantly higher mean differences in intake from non-consumers for nutrients of public health concern and other shortfall nutrients: dietary fiber; vitamins A, C, and E; calcium; magnesium; and potassium [10]. Tree nut consumers also had higher intakes of total fat; MUFA; PUFA; vitamins K and B6; thiamin; riboflavin; folate; phosphorus; iron; zinc; and copper. Tree nut consumers also had lower intakes of carbohydrates, alcohol, and sodium than non-consumers. King *et al.* [9] also showed consumers of tree nuts and all nuts had higher intakes of vitamins E, K, B_12_, and B_6_; thiamin; riboflavin; niacin; magnesium; phosphorus; copper; potassium; calcium; and iron and lower levels of sodium than non-consumers. Diet quality, as assessed by the Healthy Eating Index (HEI)-2005, was also higher in tree nut consumers than non-consumers [7,8].

Although tree nut consumption has been associated with improved nutrient intake and diet quality, previous studies [7,8,9] were limited since they used older data sets and a single 24-h dietary recall, so usual intake (UI) could not be calculated; thus nutrient adequacy could not be determined. Usual intakes represent long-term average daily intakes and remove excessive intra-person intake variation; UI are the best estimates to compare to dietary recommendations, since suggested intakes are to be met over time [11]. In addition, one of the previous studies looked only at tree nuts consumed out of hand [8] and both [7,8] determined tree nut consumption using food codes [12], rather than commodity codes [13]. The latter includes nuts used as ingredients in other foods like breads, cereals and muffins, so they are more likely to capture total intake more fully. The purposes of this study were to compare the nutrient adequacy of UI of selected nutrients and diet quality between tree nut consumers and non-tree nut consumers in a nationally representative population.

## 2. Experimental Section

### 2.1. Study Population and Analytic Sample

Data from adults 19+ years of age (year) participating in the NHANES 2005–2006, 2007–2008, and 2009–2010 were combined to increase sample size [14]. Females who were pregnant or lactating were excluded from the study. The final analytic sample (*n* = 14,386) also included only individuals with complete and reliable dietary records as determined the National Center for Health Statistics staff. The NHANES follows federal laws that ensure confidentiality and protect individual participants from identification [15]. This was a secondary data analysis which lacked personal identifiers; therefore, did not require institutional review [16].

### 2.2. Dietary Analyses

Dietary intake was determined using two multiple pass 24-h dietary recalls [17,18]. The first recall was in-person in the Mobile Examination Center [19] and the second was conducted three to ten days later via telephone [20]. The US Environmental Protection Agency-USDA Food Commodity Intake Database (FCID) commodity codes [13] were used to identify ingredients of survey foods that included all tree nuts or tree nut butters (referred to as tree nuts hereafter) made from: almonds, Brazil nuts, cashews, filberts (hazelnuts), macadamias, pecans, pistachios, walnuts, and pine nuts (almond and other nut oils used as ingredients were not included in tree nut definition). FCID complements dietary intake measured in NHANES databases in that it provides estimates of food consumption expressed as food commodities/ingredients (e.g., beef, wheat flour, tomato sauce, soybean oil) as opposed to foods items.

The gram amount of tree nut and all nuts consumed by NHANES 2005–2010 respondents was determined by applying the FCID tree nut composition data to the respondent’s 24-h recall dietary interview data. Tree nut intakes were aggregated over the entire day. Usual intake was determined using the National Cancer Institute (NCI) method [21] with survey day (one or two) and a weekend day flag (Friday/Saturday/Sunday *versus* others) as covariates. Tree nut or all nut consumers were defined by a UI of at least ¼ ounce (7.0875 grams) per day [7]. Alcohol intake (g), which was used as a covariate in the statistical analyses, was also determined via the 24-h dietary recalls.

### 2.3. Diet Quality

Diet quality was determined by calculating the total Healthy Eating Index (HEI)-2005 score and component scores [22,23]. The SAS code used to calculate HEI-2005 scores was downloaded from the Center for Nutrition Policy and Promotion website [24].

### 2.4. Statistical Analyses

Sampling weights, as provided by NHANES, were used in these analyses using SUDAAN v11.0 (Research Triangle Institute; Raleigh, NC, USA). A six-year sample weight variable was created by assigning one third of the two-year weights for 2005–2006, 2007–2008, and 2009–2010 [14,15]. Covariate adjusted least squares regression was used to determine energy intake. Usual intakes of tree nuts and selected nutrients were determined using SAS v9.2 (SAS Institute, Cary, NC, USA) via the NCI method [21]. Since tree nuts are consumed episodically, the two-part model (probability and amount) was used; for nutrients that are usually consumed daily, the one part model was used. The NCI SAS macros (Mixtran v1.1 and Distrib v1.1) generated parameter effects, after covariate adjustment, to estimate the distribution of UI via Monte Carlo simulation methods, respectively [21]. Covariates for UI determination were day of the week of the 24-h recall (coded as weekend (Friday–Sunday) or weekday (Monday–Thursday)) and the sequence of dietary recall (first or second). NCI software was used with the two days of intake using one-day sampling weights to obtain appropriate variance estimates. The Dietary Reference Intake age groups [25] were used to present UI for each of the nutrients studied.

To assess the extent of inadequate intake of nutrients with an Estimated Average Requirements (EAR), the EAR cut-point method was used (except for iron where the probability method was used) [26]. The EAR is the appropriate DRI to use when assessing the adequacy of group intakes [26]. This method provides an estimate of the proportion of individuals in the group with inadequate intakes by age and gender. For nutrients without an EAR, *i.e.*, dietary fiber, potassium, and sodium the percent above the Adequate Intake (AI) was determined [26]. To determine if there were significant differences (*p* < 0.001) for the percentage of tree nut consumers *vs.* non-consumers with intakes less than the EAR or above the AI a Z-statistic for differences in population proportions was used.

To determine whether there were differences between HEI-2005 total and component scores, least square means ± SE were compared using logistic regression. Covariates were: gender, ethnicity, age (years), socioeconomic status, physical activity level, smoking status, and alcohol intake. Alcohol intake came from 24-h dietary recalls; all other values for covariates came from questionnaire data [27].

## 3. Results

### 3.1. Study Population and Consumption

Study demographics are shown in Table 1. Tree nut consumers constituted approximately 6% of the population. Covariate adjusted mean UI of tree nuts for consumers was 44.3 ± 1.6 g/day; whereas, *per captia* consumption was 3.3 ± 0.1 g/day. Tree nut consumers were more likely to be non-Hispanic White, older, with a higher socioeconomic status, be more active/less sedentary, and less likely to be a current smoker than non-consumers.

**Table 1:** Descriptive statistics of adult tree nut consumers participating in the National Health and Nutrition Examination Survey 2005–2010.

Variable	Consumers Tree Nuts (*n* = 755)	Non-Consumers Tree Nuts (*n* = 13,631)
	Mean ± SE	Mean ± SE
**Gender—(%) Female**	50.2 ± 2.1	51.2 ± 0.5
**Ethnicity**		
Non-Hispanic White (%)	82.1 ± 1.9	70.8 ± 1.9 *
Non-Hispanic Black (%)	6.7 ± 1.2	11.6 ± 1.0 *
Mexican-American (%)	4.9 ± 0.7	8.0 ± 1.0 *
**Age (Years)**	53.1 ± 0.7	46.3 ± 0.3 *
**Poverty Index Ratio**	3.8 ± 0.06	3.0 ± 0.04 *
**Physical Activity**		
Sedentary (%)	14.9 ± 1.5	26.2 ± 0.8 *
Moderate (%)	38.2 ± 2.3	35.8 ± 0.7
Active (%)	46.9 ± 2.5	38.0 ± 0.9 *
**Smoker, Current (%)**	13.2 ± 2.1	24.0 ± 0.7 *
**Alcohol (g)**	12.1 ± 1.2	11.1 ± 0.5

* Difference between tree nut consumers and non-tree nut consumers, *p* < 0.01. Z-score comparisons were used to compare tree nuts consumers and non-consumers.

### 3.2. Usual Intake and Nutrient Adequacy

Energy intake was significantly higher for tree-nut consumers (2247 ± 16 kcals) than in non-consumers (2042 ± 17 kcals). Usual intake data for select micronutrients are presented in Table 2 and Table 3. These data showed that tree nut consumers had a lower percentage (*p* < 0.0001) of the population with UI below the EAR for vitamins A (21.6 ± 5.1 *vs.* 48.8 ± 1.0), E (37.7 ± 3.8 *vs.* 94.2 ± 0.4), and C (17.3 ± 4.0 *vs.* 44.2 ± 1.0); folate (2.5 ± 1.5 *vs.* 12.0 ± 0.6); calcium (26.9 ± 2.5 *vs.* 44.3 ± 0.7); iron (3.1 ± 0.6 *vs.* 8.6 ± 0.4); magnesium (8.2 ± 1.4 *vs.* 60.1 ± 0.8); and zinc (1.5 ± 1.0 *vs.* 12.8 ± 0.8) (Table 2). Consumers of tree nuts were also more likely to have UI above (*p* < 0.0001) the AI for dietary fiber (33.3 ± 2.9 *vs.* 3.7 ± 0.3) and potassium (11.9 ± 2.5 *vs.* 1.9 ± 0.2) (Table 3). Almost all consumers and non-consumers had sodium intakes in excess of the AI.

**Table 2:** Usual intake and percentage of the population below the estimated average requirement in tree nut consumers compared with non-tree nut consumers.

Variable	Consumer Status	Usual Intake		Percentile					EAR	
		Mean	SE	10	25	50	75	90	% Below	SE
Vitamin A RAE (mcg)	Consumer	796	29	458	588	759	963	1181	21.6 *	5.1
Vitamin A RAE (mcg)	Non-Consumer	610	6	298	408	564	761	980	48.8	1.0
Vitamin D (mcg) ^1^	Consumer	5.9	0.4	2.3	3.5	5.3	7.7	10.3	88.8	3.2
Vitamin D (mcg) ^1^	Non-Consumer	4.5	0.0	1.8	2.6	4.0	5.8	8.0	95.9	0.3
Vitamin E (mg) ^2^	Consumer	14.0	0.4	8.6	10.6	13.4	16.7	20.4	37.7 *	3.8
Vitamin E (mg) ^2^	Non-Consumer	7.3	0.1	4.1	5.3	6.9	8.8	10.9	94.2	0.4
Folate, DFE (mcg)	Consumer	655	19	400	489	616	777	962	2.5 *	1.5
Folate, DFE (mcg)	Non-Consumer	537	5	307	391	508	652	807	12.0	0.6
Vitamin B12 (mcg)	Consumer	6.4	0.3	3.0	4.2	5.9	8.0	10.6	2.3	1.0
Vitamin B12 (mcg)	Non-Consumer	5.3	0.05	2.7	3.6	4.8	6.5	8.4	3.1	0.3
Vitamin C (mg)	Consumer	116	5	55.3	76.6	107	145	188	17.3 *	4.0
Vitamin C (mg)	Non-Consumer	83.5	1.2	31.3	47.5	72.7	107.3	149.2	44.2	1.0
Calcium (mg)	Consumer	1136	23	712	873	1091	1345	1620	26.9 *	2.5
Calcium (mg)	Non-Consumer	954	6	536	688	898	1158	1444	44.3	0.7
Iron (mg)	Consumer	19.5	0.5	12.1	14.7	18.4	23.1	28.3	3.1 *	0.6
Iron (mg)	Non-Consumer	15.3	0.1	9.2	11.5	14.6	18.3	22.5	8.6	0.4
Magnesium (mg)	Consumer	467	8	311	367	443	542	655	8.2 *	1.4
Magnesium (mg)	Non-Consumer	290	2	181	223	279	345	415	60.1	0.8
Zinc (mg)	Consumer	15.5	0.5	9.8	11.6	14.4	18.1	22.6	1.5 *	1.0
Zinc (mg)	Non-Consumer	11.9	0.1	7.1	8.8	11.2	14.3	17.7	12.8	0.8

Source: NHANES, 2005–2010, 19+ years of age, excluding pregnant/lactating females. Regression analyses with the following covariates: day of the week of the 24-h recall (coded as weekend (Friday–Sunday) or weekday (Monday–Thursday)) and the sequence of dietary recall (first or second) were used to determine the differences between groups. Abbreviations: EAR = estimated average requirement; RAE = retinol activity equivalent; DFE = dietary folate equivalent ^1^ Vitamin D (D2 + D3); ^2^ Vitamin E as alpha-tocopherol. Difference between consumers and non-consumers significant, *p* < 0.001.

**Table 3:** Usual intake and percentage of the population above the adequate intake in tree nut consumers compared with non-tree nut consumers.

Variable	Gender	Usual Intake		Percentile					AI	
		Mean	SE	10	25	50	75	90	% Above	SE
Dietary fiber (gm)	Consumer	24.2	0.7	14.9	18.3	22.9	28.5	35.2	33.3 *	2.9
Dietary fiber (gm)	Non-Consumer	15.7	0.1	9.0	11.6	15.0	19.1	23.4	3.73	0.3
Potassium (mg)	Consumer	3506	66	2373	2802	3363	4065	4837	11.9 *	2.5
Potassium (mg)	Non-Consumer	2662	13	1669	2058	2565	3165	3787	1.9	0.2
Sodium (mg)	Consumer	3817	71	2531	2982	3610	4443	5387	99.99	0.03
Sodium (mg)	Non-Consumer	3597	21	2241	2738	3425	4292	5200	99.7	0.1
Total Choline (mg)	Consumer	393	8	257	303	371	463	560	15.4	2.6
Total Choline (mg)	Non-Consumer	327	2	195	242	308	394	486	5.9	0.4

Source: NHANES, 2005–2010, 19+ years of age, excluding pregnant/lactating females. Regression analyses with the following covariates: day of the week of the 24-h recall (coded as weekend (Friday–Sunday) or weekday (Monday–Thursday)) and the sequence of dietary recall (first or second) were used to determine the differences between groups. Abbreviations: AI = adequate intake. Difference between consumers and non-consumers significant, *p* < 0.001.

### 3.3. Diet Quality

The HEI-2005, an objective measure of diet quality developed and validated by USDA, was significantly (*p* < 0.0001) higher in tree-nut consumers (61.2 ± 0.7 *vs.* 52.4 ± 0.3) compared to non-tree nut consumers. In addition, the total fruit (*p* = 0.0002), whole fruit (*p* = 0.0002), dark green and orange vegetables and legumes (*p* = 0.0002), meat and beans (*p* < 0.0001), whole grains (*p* = 0.0041), sodium (*p* < 0.0001), and Solid Fat, Alcohol, and Added Sugars kilocalories (*p* < 0.0001) component scores were higher in tree nut consumers compared with non-tree nut consumers. Component score of total grains was lower (*p* < 0.0001) in tree nut consumers than in non-consumers (Table 4).

**Table 4:** Least-square means of Healthy Eating Index-2005 (HEI-2005) component scores by consumption of tree nuts in adults 19+ years of age.

HEI-2005 Component Score	Tree-Nut Consumers	Tree Nut Non-Consumers
	LS Mean ± SE	LS Mean ± SE
Total HEI-2005 Score (100)	61.2 ± 0.7 *	52.4 ± 0.3
Total Fruit (5)	2.5 ± 0.1 *	2.1 ± 0.03
Whole Fruit (5)	2.4 ± 0.1 *	2.0 ± 0.03
Total Vegetables (5)	3.1 ± 0.1	3.0 ± 0.03
Dark Green & Orange Veg & Legumes (5)	1.7 ± 0.1 *	1.2 ± 0.03
Meat and Beans (5)	9.2 ± 0.1 *	8.0. ± 0.04
Total Grains (5)	3.7 ± 0.1 *	4.2 ± 0.02
Whole Grains (5)	1.4 ± 0.1 *	1.1 ± 0.02
Milk (10)	5.1 ± 0.1	5.2 ± 0.05
Oils (10)	8.7 ± 0.1 *	6.3 ± 0.05
Saturated Fat (10)	6.2 ± 0.2	5.8 ± 0.1
Sodium (10)	4.4 ± 0.2 *	3.3 ± 0.04
SoFAAS Calories (20)	12.8 ± 0.3 *	10.2 ± 0.1 *

Source: NHANES, 2005–2010, 19+ years of age, excluding pregnant/lactating females. Sample-weighted least-square (LS) mean and standard error (SE) are estimated using PROC REGRESS of SUDAAN. Covariates include gender, ethnicity, age (years), socioeconomic status, physical activity level, smoker status, and alcohol intake. Difference between consumers and non-consumers significant, *p* < 0.01.

## 4. Discussion

Previous studies showed tree nut consumption was associated with better nutrient intake [7,8,9]. The current study extended these studies by looking at nutrient adequacy. This study showed that based on the UI of tree nut consumption for selected nutrients, nutrient adequacy was better in tree nut consumers than in non-tree nut consumers. Despite the relatively low percentage of individuals consuming tree nuts shown, the weighted number of tree nut consumers actually represented over 12,440,000 individuals, not an insignificant number. This study also confirmed that tree nut consumption was associated with better diet quality than that seen in non-tree nut consumers [7,8].

This study is similar to two previously published articles [7,8]; however, those studies used NHANES data from 1999–2004. Previous work [7,8] showed that tree nut consumption led to small, but significant positive changes in nutrient intake and diet quality; therefore, it was important to reconfirm these findings, especially since new data from approximately 15,000 adults have become available.

One difference between this study and two previous ones is that it used the FDIC commodity codes [13] to determine intake rather than just nut based food codes [12]. The advantage of this is that the FDIC database provides estimates of food consumption “as eaten,” that is in terms of ingredients, which can provide more utility.

Assessment of nutrient adequacy is a step forward from previous studies of nutrient intake among tree nut consumers, since it is a better estimate of how groups [25] meet the EAR and AI than does mean nutrient intake. To determine nutrient adequacy, an individual’s UI must be determined, so more than one dietary recall must be available. The NHANES did not begin releasing two days of 24 hour dietary recalls until 2003. Thus, although participants in the last NHANES data set 2003–2004 could have had UI calculated, the sample size may not have been statistically adequate to compare data from tree nut consumers. Another potential caveat of determining UI is that it requires a large number of diet recalls assessed using accurate food composition information; the NHANES can provide suitable sample sizes if cycle releases are concatenated to increase size. This is the first manuscript that has examined the association between tree nut consumption and dietary adequacy.

The 2010 Dietary Guidelines for Americans [10] recognizes that although a variety of healthful foods are available in the US, many do not eat nutrient rich foods and, thus, intakes of several micronutrients are low. Thus, intake of dietary fiber and some micronutrients including vitamin D, calcium, and potassium, are low enough to be classified as nutrients of public health concern [10]. Low intake of several other nutrients is also of concern for specific population groups, such as folic acid for women of child-bearing potential, iron, and vitamin B_12_. The DGA has also identified other nutrients, including vitamins A, C, and K, and magnesium [10] as underconsumed in the US. Vitamin E was identified as a shortfall nutrient by the 2005 Dietary Guidelines Advisory Committee [28]; however, although intake remains low [29], no recommendations were made by the 2010 DGA.

A higher percentage of individuals consuming tree nuts were above the EAR for vitamins A, C, and E; folate; calcium; iron; magnesium; and zinc, than non-tree nut consumers. Of the nutrients examined, only vitamins D and B_12_ were not associated with better nutrient adequacy in tree nut consumers; this was not surprising since tree nuts are poor sources of these nutrients [2].

Health benefits of vitamins A and C, folate, calcium, and magnesium are well established. Vitamin A deficiency is well known for its association with xerophthalmia, depressed immune function and resistance to infectious diseases, anemia, and increased morbidity and mortality in pregnant females. Vitamin A is an underconsumed nutrient in the US [10]; on average, adult females consume approximately one-third of the daily value, and adult males consume approximately 40% of the daily value of vitamin A [30]. Vitamin C is critical for the biosynthesis of collagen and as a co-factor in the biosynthesis of catecholamines, cholesterol, amino acids, and some peptide hormones [31]. Vitamin C deficiency is well known for its association with scurvy [31]. Clinical scurvy is rare in developed countries; however, it is still found in undernourished individuals, including those with low-incomes, the elderly, the chronically ill, and alcoholics [32].

Folate, is inversely associated with the risk of neural tube defects [33]; however; females of child-bearing age continue to have intakes of folate below recommendations [34,35]. Calcium is usually associated with the structure of bone and teeth and nerve conduction. It is important to consume foods that are associated with higher levels of nutrient adequacy of folate and calcium, such as tree nuts. Magnesium is an essential cofactor for more than 350 enzymatic reactions, especially those involving high-energy phosphate bonds [36]. Higher levels of magnesium intake have been inversely associated cardiovascular disease, hypertension, type 2 diabetes mellitus, metabolic syndrome, and osteoporosis [37].A significantly lower percentage of tree nut consumers were below the EAR for the above nutrients when compared with non-tree nut consumers, suggesting that consumption of tree nuts or foods consumed with them can help to assuage this problem.

Vitamin E was not named as an underconsumed nutrient by the 2010 DGA, although it was identified as a shortfall nutrient by the 2005 DGA; reasons for the difference are not clear. Vitamin E is a peroxyl radical scavenger that protects the PUFA in membranes from oxidation [38]. That tree nut consumption is associated with a lower prevalence of those below the EAR for vitamin E is especially important since less than 10% of the population meets the EAR for vitamin E intake from food [39]. Vitamin E is the most potent antioxidant in plasma, and is associated with reduced risk of heart disease [40], type 2 diabetes [41], hypertension [42], and all-cause mortality [43]. Tree nut consumption may be a way to improve vitamin E intake through food while keeping within the recommendations for dietary fat.

A higher percentage of tree nut consumers was above the AI for most nutrients without an EAR, especially, dietary fiber and potassium. Over 30% of tree nut consumers had intakes above the AI for dietary fiber and only 3.7% of non-consumers were above the AI. However, dietary fiber intake [29] is still largely below the recommended intakes [24]. Dietary fiber intake is associated with many well-known health benefits including improved weight status, serum cholesterol levels, blood pressure, and blood sugar control [44]. Tree nuts provide an easy way to include dietary fiber into the diet. However, the dietary fiber content of tree nuts varies; for example, a single ounce provides from 0.9 gm fiber (cashews) to 3.5 gm (almonds) [2]. The fiber intake of tree nut consumers was higher than the fiber content of the mean level of tree nuts in the diet, suggesting that other high fiber foods are contributing to overall intake and that tree nut consumers may have an overall healthier diet than non-consumers. The HEI-2005 component score results also suggest that since tree nut consumers had higher scores of whole and total fruit, dark green and orange vegetables and legumes, meat and beans, and total and whole grains, these foods may also have provided fiber to the diet.

Potassium intake is thought to be low because of the low intake of fruit, vegetables, and dairy [10]—all of which are high potassium foods. Higher intakes of potassium could reduce the risk of hypertension and thus the risk of cardiovascular disease and stroke [45], in part by blunting the effect of dietary sodium [46,47]. In 2009–2010, the overall age-adjusted prevalence of hypertension among US adults was 28.6% [48]. Most species of tree nuts are good sources of potassium; for example almonds, pine nuts, pecans, and have 200, 169, and 116, mg/oz of potassium, respectively.

There was no difference in usual sodium intake between those above the AI in those consuming or not consuming tree nuts. Mean UI exceeded recommendations [24] in both groups. Previous studies [7,8] have shown that although sodium intake was high in tree nut consumers and non-consumers, consumers had higher intakes of sodium. Since tree nuts themselves are very low in sodium with an average content of 2 mg/10.5 g. It is possible that the foods consumed with tree nuts contributed high levels of sodium or consumers were consuming salted nuts rather than plain roasted tree nuts; however, the database does not distinguish between salted and unsalted tree nuts. Differences in methodology in assessing dietary intake or secular trends in the type of nuts consumed may have led to the differences with previous studies.

Strengths of this study include that it encompassed a large, recent, nationally representative sample achieved through combining several sets of NHANES data released and used the NCI method to assess UI and the percentage of the population below recommended levels for nutrients in tree nut and non-tree nut consumers. *Limitations* of the study include the use of 24-h dietary recalls, which have several inherent limitations. Participants relied on memory to self-report dietary intakes; therefore, data were subject to non-sampling errors, including underreporting of energy and examiner effects. Respondents may not have differentiated between tree nuts and ground nuts (peanuts), which are often served together and may be perceived by the general public to be “nuts”. Peanuts were specifically not included in this study since we felt that with the more than four times increase in utilized production of tree nuts since 1980 [49], it was important to study tree nuts as a separate food category. Supplement intake was not considered in this study, since it is the “fundamental premise” of the 2010 Dietary Guidelines Advisory Committee [50] that nutrients should come from food. Multicollinearity of diet could also have accounted for the differences in nutrient intake between tree nut and non-tree nut consumers. This concept is especially important when conducting cross-sectional analyses since causal inferences cannot be drawn.

## 5. Conclusions

Tree nut consumption was associated with better nutrient adequacy and diet quality than those seen among non-consumers. Tree nut consumption should be encouraged by health professionals, including registered dietitians, as an overall part of a total diet [51]. Nutrition education programs that increase awareness and consumption of tree nuts should be designed and delivered. Nutrition education programs should encourage consumption of tree nuts without the addition of excess sodium.
